# Serum- and glucocorticoid-inducible kinase 1 and the response to cell stress

**DOI:** 10.15698/cst2019.01.170

**Published:** 2018-12-02

**Authors:** Florian Lang, Christos Stournaras, Nefeli Zacharopoulou, Jakob Voelkl, Ioana Alesutan

**Affiliations:** 1 Department of Vegetative and Clinical Physiology, Eberhard-Karls-University, Tübingen, Germany.; 2 Department of Biochemistry, University of Crete Medical School, Voutes, Heraklion, Greece; 3 Department of Internal Medicine and Cardiology, Charité – Universitätsmedizin Berlin, Germany.; 4 DZHK (German Centre for Cardiovascular Research), partner site Berlin, Germany.; 5 Berlin Institute of Health (BIH), Berlin, Germany.

**Keywords:** SGK1, glycolysis, cell survival, cell migration, angiogenesis, fibrosis and tissue calcification

## Abstract

Expression of the serum- and glucocorticoid-inducible kinase 1 (SGK1) is up-regulated by several types of cell stress, such as ischemia, radiation and hyperosmotic shock. The SGK1 protein is activated by a signaling cascade involving phosphatidylinositide-3-kinase (PI3K), 3-phosphoinositide-dependent kinase 1 (PDK1) and mammalian target of rapamycin (mTOR). SGK1 up-regulates Na^+^/K^+^-ATPase, a variety of carriers including Na^+^-,K^+^-,2Cl^−^- cotransporter (NKCC), NaCl cotransporter (NCC), Na^+^/H^+^ exchangers, diverse amino acid transporters and several glucose carriers such as Na^+^-coupled glucose transporter SGLT1. SGK1 further up-regulates a large number of ion channels including epithelial Na^+^ channel ENaC, voltagegated Na^+^ channel SCN5A, Ca^2+^ release-activated Ca^2+^ channel (ORAI1) with its stimulator STIM1, epithelial Ca^2+^ channels TRPV5 and TRPV6 and diverse K^+^ channels. Furthermore, SGK1 influences transcription factors such as nuclear factor kappa-B (NF-κB), p53 tumor suppressor protein, cAMP responsive element-binding protein (CREB), activator protein-1 (AP-1) and forkhead box O3 protein (FOXO3a). Thus, SGK1 supports cellular glucose uptake and glycolysis, angiogenesis, cell survival, cell migration, and wound healing. Presumably as last line of defense against tissue injury, SGK1 fosters tissue fibrosis and tissue calcification replacing energy consuming cells.

## INTRODUCTION

The ubiquitously expressed [1–4] serum- and glucocorticoid-inducible kinase 1 (SGK1) has originally been cloned as a gene up-regulated by serum and glucocorticoids in rat mammary tumor cells [1, 5]. The human SGK1 has been identified as a gene up-regulated by cell shrinkage [6].

### SGK1 expression

Expression of SGK1 is highly variable and subject to regulation by a wide variety of triggers including hyperosmotic or isotonic cell shrinkage, dehydration, excessive glucose concentrations, mechanical stress, oxidative stress, heat shock, radiation, DNA damage, ischemia, neuronal injury and neuronal excitation [1, 3, 7–12]. SGK1 transcription is further up-regulated by several hormones and mediators including glucocorticoids, mineralocorticoids, gonadotropins, gestagens, 1,25(OH)_2_D_3_, erythropoietin, morphine, transforming growth factor β (TGFβ), interleukin-6, fibroblast and platelet-derived growth factor, thrombin, endothelin, advanced glycation end products (AGEs) and activation of peroxisome proliferator-activated receptor γ (PPARγ) [1]. Inhibitors of SGK1 expression include serum starvation, heparin, dietary iron, nucleosides and nephrilin [1]. Overall, SGK1 expression declines with age [13].

Signaling of transcriptional SGK1 regulation involves cytosolic Ca^2+^, cyclic AMP, stress-activated protein kinase-2 (SAPK2 or p38 MAPK kinase), protein kinase C (PKC), protein kinase RAF, big mitogen-activated protein kinase 1 (BMK1, also known as extracellular signal-regulated kinase ERK5), extracellular signal-regulated kinase 1/2 (ERK1/2), phosphatidylinositide-3-kinase (PI3K), reactive oxygen species, NADPH oxidases, nitric oxide and EWS/NOR1 (NR4A3) fusion protein [1].

The SGK1 promoter binds receptors for glucocorticoids (GR), mineralocorticoids (MR), progesterone (PR), 1,25(OH)_2_D_3_ (VDR), retinoids (RXR), farnesoids (FXR), sterol regulatory element-binding protein (SREBP), PPARγ, cAMP response element-binding protein (CREB), p53 tumor suppressor protein, Sp1 transcription factor, activator protein 1 (AP-1), activating transcription factor 6 (ATF6), heat shock factor (HSF), reticuloendotheliosis viral oncogene homolog (c-Rel), nuclear factor kappa- B (NF-kB), signal transducers and activators of transcription (STAT), TGFβ-dependent transcription factors SMAD3 and SMAD4, forkhead activin signal transducer (FAST) and the transcription factor TonE binding protein (TonEBP or NFAT5) [1].

SGK1 translation is stimulated by PI3K and requires actin polymerization [14].

### SGK1 activation and its degradation

Once expressed SGK1 requires activation. Stimulators of SGK1 activity include insulin, IGF1, hepatic growth factor (HGF), follicle stimulating hormone (FSH), thrombin and corticosterone [1]. Signaling involving activation of SGK1 includes PI3K and 3-phosphoinositide (PIP3)-dependent kinase PDK1 [6]. Interaction of SGK1 and PDK1 is supported by the scaffold protein Na^+^/H^+^ exchanger regulating factor 2 (NHERF2) [3]. PIP3 is degraded and activation of SGK1 thus suppressed by the phosphatase and tensin homolog PTEN [3]. SGK1 activation further involves WNK1 (lysine deficient protein kinase 1) and mammalian target of rapamycin mTOR complex-2 (mTORC2) composed of mTOR, Rictor (rapamycin-insensitive companion of mTOR), Sin1 (stress-activated protein kinase-interacting protein 1), mLST8 and Protor-1 [1, 15–27]. SGK1 is further up-regulated by p38α MAPK, ERK5, cAMP, lithium, Ca^2+^-sensitive calmodulin-dependent protein kinase kinase (CaMKK), G-protein Rac1, neuronal depolarization, oxidation, hypertonicity, and fibronectin [1, 3, 6, 28].

SGK1 degradation is triggered by ubiquitination involving NEDD4-2 (neuronal precursor cells expressed developmentally down-regulated) [1, 3] and Rictor/Cullin-1 [1, 29–31]. SGK1 degradation is inhibited by glucocorticoid-induced leucine zipper protein-1 (GILZ) [32].

### SGK1 kinase targets

The optimal consensus sequences for phosphorylation by SGK1 are R-X-R-X-X-(S/T)-phi and R-R-X-S/T (X = any amino acid, R = arginine, S = serine, T = threonine, phi = hydrophobic amino acid) [3, 33]. Specific SGK1 targets are N-myc down-regulated genes NDRG1 and NDRG2 [1, 3]. Other SGK1 targets are shared by other kinases including SGK and protein kinase B (PKB/Akt) isoforms [3].

SGK1 influences a variety of enzymes including ubiquitin ligase NEDD4-2, inducible nitric oxide synthase iNOS, phosphomannose mutase 2 (PMM2), phosphatidylinositol-3-phosphate-5-kinase (PIKfyve), serine/threonine kinase WNK4, ERK2 (MAPK1), mitogen-activated protein kinase/ERK kinase kinase 3 (MEKK3), stress-activated kinase (SEK1), B-Raf kinase, glycogen synthase kinase 3 (GSK-3), p53-ubiquitinating MDM2 and Notch1-IC protein degradating Fbw7 [1].

SGK1 up-regulates transcription factors such as CREB, AP-1 and NF-κB [1, 34–37]. On the other hand, SGK1 phosphorylates and thus activates NDRG1, which in turn down-regulates NF-κB signaling [1, 38]. Moreover, SGK1 down-regulates transcription factor p53 and forkhead box O3 protein (FOXO3a) [1, 39, 40].

SGK1 is a powerful regulator of several ion channels [1, 3, 41], including epithelial Na^+^ channel ENaC, voltage-gated Na^+^ channel SCN5A, renal outer medullary K^+^ channel ROMK1, voltage-gated K^+^ channels KCNE1/KCNQ1, KCNQ4, Kv1.3, Kv1.5, Kv7.2/3, Kv4.3 and hERG, the Ca^2+^ release-activated Ca^2+^ channel ORAI1 and its stimulator STIM1, transient receptor potential channels TRPV4, TRPV5 and TRPV6, kainate receptor GluR6, unselective cation channel 4F2/LAT, Cl^-^ channels ClCka/barttin, ClC2, CFTR (Cystic fibrosis transmembrane conductance regulator) and VSOAC (volume-sensitive osmolyte and anion channel) as well as acid-sensing ion channel ASIC1 [1, 3].

SGK1 stimulates diverse carriers including Na^+^-,K^+^-,2Cl^–^- cotransporter NKCC2, NaCl cotransporter NCC, Na^+^/H^+^ exchangers NHE1 and NHE3, glucose carriers SGLT1, GLUT1 and GLUT4, amino acid transporters ASCT2, SN1, B(0)AT1, EAAT1, EAAT2, EAAT3, EAAT4 and EAAT5, peptide transporters PepT, Na^+^,dicarboxylate cotransporter NaDC-1, creatine transporter CreaT, Na^+^,myoinositol cotransporter SMIT as well as phosphate carriers NaPiIIa and NaPiIIb [1, 3]. Furthermore, SGK1 up-regulates the Na^+^/K^+^-ATPase and albumin uptake [1, 3].

Further targets of SGK1 include nephrin, type A natriuretic peptide receptor (NPR-A), Ca^2+^-regulated heat-stable protein of apparent molecular mass 24 kDa (CRHSP24), the adaptor precursor (APP) Fe65, NDRG1 and NDRG2, myosin-Vc, filamin C, microtubule-associated protein tau, Cyclin-dependent kinase inhibitor 1B (*p27*^Kip1^), and huntingtin [1, 3, 40, 42–44].

The present review discusses the role of SGK1 in the orchestration of cellular response to stress such as energy depletion. The case is made that SGK1 supports cellular energy supply by stimulation of glucose uptake and glycolysis, as well as by stimulation of angiogenesis. SGK1 supports cell survival and cell migration, a prerequisite of tissue repair. As last line of defense, SGK1 replaces energy consuming cells with extracellular matrix by stimulation of tissue fibrosis and tissue calcification. In order to limit the number of citations some of the earlier original papers have been replaced by reviews.

## GLUCOSE UPTAKE AND GLYCOLYSIS

SGK1 stimulates cellular glucose uptake and thus enhances the availability of glucose for glycolysis [3]. SGK1 further stimulates the Na^+^/H^+^ ion exchanger [36] which generates an alkaline cytosolic pH, a prerequisite for an increase of glycolytic flux [1]. The up-regulation of SGK1 in ischemia thus supports energy supply by glycolysis [2, 3, 10, 45].

## ANGIOGENESIS

SGK1 is required for angiogenesis during embryonic development [46] and following ischemia in the adult [47]. In myocardial ischemia, lack of SGK1 blunts the phosphorylation of SGK1 target protein NDRG1 and compromises the up-regulation of transcription factor NF-κB and its target protein, VEGF-A (vascular endothelial growth factor A). Lack of SGK1 further impairs endothelial cell (ECs) migration and tube formation *in vitro*, and decreases *in vivo* angiogenesis after myocardial infarction [47].

## CELL SURVIVAL

SGK1 supports cell survival and cell proliferation of both tumor cells and neurons [1, 3, 7, 10, 48–52]. SGK1 is highly expressed in several tumors [10], including non-small cell lung cancer [53], colon cancer [10], prostate cancer [54], ovarian tumors [1], myeloma [55], and medulloblastoma [1]. SGK1 confers resistance of breast cancer cells to chemotherapy [3, 10, 56], and inhibition of SGK1 sensitizes tumor cells to cytotoxic drugs or radiation [12]. SGK1 contributes to androgen-induced growth of prostate cancer cells [2]. SGK1 counteracts the pro-apoptotic effect of membrane androgen receptors (mAR) [1] in colon carcinoma cells [57–59]. Lack of SGK1 blunts the development of spontaneous tumors in APC-deficient mice [2] and chemically-induced colonic tumors in wild-type mice [1].

SGK1 stimulates cell proliferation and inhibits cell death in part by up-regulating channels and transporters, such as the store-operated Ca^2+^ entry (SOCE) accomplished by ORAI1/STIM1 [1, 12, 34, 35, 60, 61]. SOCE maintains oscillations of cytosolic Ca^2+^ activity, which are required for depolymerization of the actin filament network, a prerequisite for cell proliferation [3, 10]. Ca^2+^ entry is driven by the cell membrane potential, which is generated by SGK1 sensitive K^+^ channels [3, 10]. The protective effect of SGK1 on neurons similarly involves, at least in part, up-regulation of ORAI1/STIM1 [51].

SGK1 further inactivates the pro-apoptotic forkhead transcription factor FOXO3A/ FKRHL1 [1], inhibits GSK-3 and up-regulates oncogenic β-catenin [3, 7], activates IKKβ with subsequent phosphorylation and degradation of the inhibitory protein IκB and translocation of NF-κB into the nucleus [10], activates the ubiquitin ligase MDM2 with subsequent MDM2-dependent ubiquitination and proteosomal degradation of pro-apoptotic transcription factor p53 [1], disrupts binding of SEK1 to JNK1 and MEKK1 [3, 10] and up-regulates Ran binding protein (RanBP), an effect affecting microtubule network and blunting taxol sensitivity of cancer cells [52, 62].

## CELL MIGRATION

SGK1 is part of the machinery stimulating cell migration [47, 57, 58, 63, 64]. As shown in vascular smooth muscle cells (VSMCs) [64], the stimulation of migration by platelet-derived growth factor PDGF is paralleled by up-regulation of both, SGK1 expression and SGK1 activity [65, 66]. Genetic knockout of SGK1 decreases migration [64]. SGK1 is effective, at least in part, by up-regulation of the store-operated Ca^2+^ entry (SOCE), which is accomplished by the Ca^2+^ channel ORAI1 and its regulator STIM1. Expression of ORAI1 and STIM1 is stimulated by NF-κB, a transcription factor up-regulated by SGK1 [1, 64]. In VSMCs, SGK1 triggers nuclear translocation of transcription factor NF-κB [64].

## INFLAMMATION AND FIBROSIS

SGK1 contributes to the orchestration of inflammation [52, 67–70]. The kinase is required for the interleukin-23 (IL-23)-sensitive generation of interleukin-17 (IL-17)-producing CD4^+^ helper T cells (T_H_17 cells) [71]. T_H_17 cells up-regulate the pro-inflammatory cytokines GM-CSF, TNF-α and interleukin-2 (IL-2) [71].

SGK1 further contributes to fibrosis in several clinical conditions, including lung fibrosis, diabetic nephropathy, glomerulonephritis, experimental nephrotic syndrome, obstructive nephropathy, cardiac remodeling, liver cirrhosis, fibrosing pancreatitis, peritoneal fibrosis, Crohn's disease and coeliac disease [1, 3, 72–75]. The expression of SGK1 is upregulated by TGFβ [3], a pivotal stimulator of fibrosis [69, 76–81]. Signaling of TGFβ includes activation of transcription factors SMAD2/3 [1], which are ubiquitinated and, thus, tagged for degradation by NEDD4L [1]. The ubiquitin ligase is inactivated by SGK1 which thus augments TGFβ action [1]. SGK1 supports inflammation and fibrosis further by activating NF-κB [3], a proinflammatory and profibrotic transcription factor [1, 82, 83]. NF-κB up-regulates connective tissue growth factor (CTGF), which in turn contributes to stimulation of cardiac remodeling and fibrosis [1, 3, 84–87], renal proteinuria and failure [88], skin aging [15], as well as fibronectin formation at hyperglycemia [1].

## VASCULAR CALCIFICATION

SGK1 further participates in the orchestration of medial vascular calcification [84], which results mainly from osteo-/chondrogenic transdifferentiation of VSMCs [84]. Various triggers of VSMC osteo-/chondrogenic transdifferentiation induce a sharp increase of SGK1 expression [84]. Upregulation of SGK1 was also observed in the vasculature of rats with renal failure [89]. SGK1 increases the expression of the osteo-/chondrogenic transcription factors *MSX2* and *CBFA1*, which in turn stimulate the expression of alkaline phosphatase *ALPL* [84]. The enzyme fosters vascular calcification by degrading the endogenous calcification inhibitor pyrophosphate. The effect of SGK1 on osteo-/chondrogenic transdifferentiation depends on transcriptional activity of NF-κB, a decisive regulator of vascular calcification [90, 91]. NF-κB also reduces pyrophosphate release via tristetraprolin (TTP)-mediated destabilization of ankylosis protein homolog (ANKH) mRNA [90, 91].

## THE ROLE OF SGK1 IN DISEASE – CLINICAL IMPLICATIONS

A wide variety of observations point to a role of SGK1 in human pathophysiology [12]. Excessive expression and activity of SGK1 participates in the pathophysiology of diverse disorders, such as hypertension, obesity, diabetes, thrombosis, stroke, fibrosing disease, vascular calcification, infertility, autoimmune disease, and tumor growth [12,71,84]. A SGK1 gene variant (prevalence approx. 3-5% in Caucasians and approx. 10% in Africans) is associated with hypertension, stroke, obesity and type 2 diabetes [12]. Little is known about the clinical impact of SGK1 deficiency. In a SV129 genetic background, the phenotype of SGK1 knockout mice is mild and SGK1-dependent functions are apparently in large part maintained by other kinases [12]. In view of the putative role of SGK1 in neuronal survival [51], however, the possibility must be kept in mind that lack of SGK1 may accelerate the clinical course of neurodegeneration. Clearly, additional experimental and observational effort is required to define the pathophysiological impact of deranged SGK1 activity in human disease.

## CONCLUSIONS

**Figure 1:**
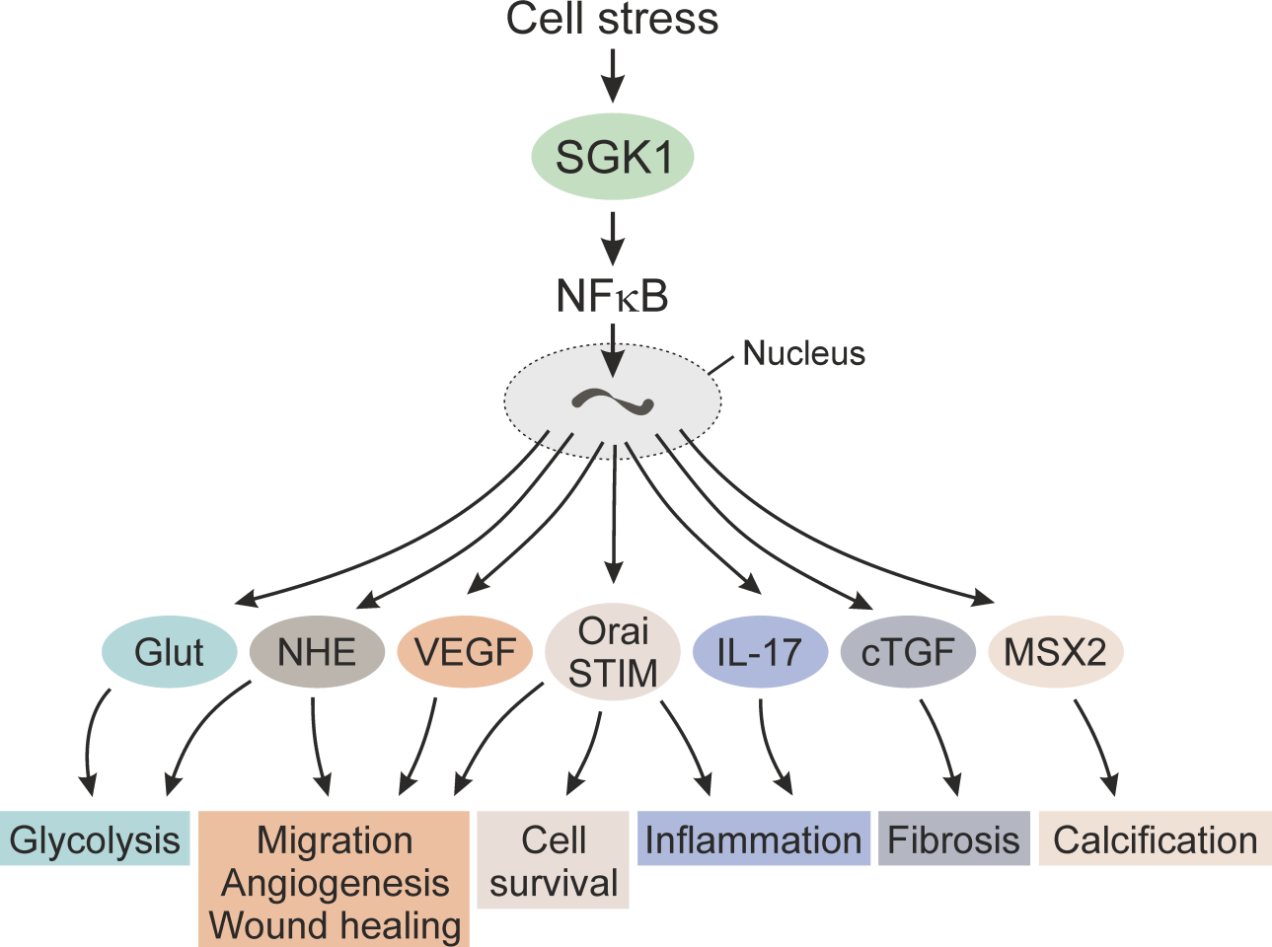
FIGURE 1: SGK1-sensitive NFκB-dependent transcription in the response to cell stress. Please note that additional NFκB-dependent genes as well as NFκB-independent mechanisms contribute to the cellular response to stress.

Expression of the serum- and glucocorticoid-inducible kinase SGK1 is steeply up-regulated following cell stress, such as ischemia, radiation and hyperosmotic shock. The SGK1 protein is activated by a signaling cascade involving phosphatidylinositide-3-kinase (PI3K), 3-phosphoinositide-dependent kinase 1 (PDK1) and mTOR. SGK1 is a powerful stimulator of transport across the cell membrane, such as Na^+^/K^+^-ATPase, Na^+^/H^+^ exchangers, cellular glucose uptake and ORAI1/STIM1-dependent store-operated Ca^2+^ entry (SOCE). SGK1 is further a powerful stimulator of transcription factors including nuclear factor κB (NF-κB; **Figure 1**). Upon cell stress such as energy depletion, SGK1 supports cellular glucose uptake and glycolysis, angiogenesis, cell survival, cell migration, and wound healing. If those functions fail to remove the cell stress, SGK1 initiates replacement of energy consuming cells by fibrotic and/or calcified tissue.

## Abbreviations:

mTOR: mammalian target of rapamycin
NF-κB: nuclear factor-kappa B
SGK1: serum- and glucocorticoid-inducible kinase 1
TGFβ: transforming growth factor beta
VSMC: vascular smooth muscle cell
